# Discovery and characterization of two new stem rust resistance genes in *Aegilops sharonensis*

**DOI:** 10.1007/s00122-017-2882-8

**Published:** 2017-03-08

**Authors:** Guotai Yu, Nicolas Champouret, Burkhard Steuernagel, Pablo D. Olivera, Jamie Simmons, Cole Williams, Ryan Johnson, Matthew J. Moscou, Inmaculada Hernández-Pinzón, Phon Green, Hanan Sela, Eitan Millet, Jonathan D. G. Jones, Eric R. Ward, Brian J. Steffenson, Brande B. H. Wulff

**Affiliations:** 1 John Innes Centre, Norwich Research Park, Norwich, NR4 7UH UK; 2 The Sainsbury Laboratory, Norwich Research Park, Norwich, NR4 7UH UK; 3 Department of Plant Pathology, University of Minnesota, Saint Paul, MN 55108 USA; 4 Institute for Cereal Crops Improvement, Tel Aviv University, Tel Aviv, 69978 Israel; 5 2Blades Foundation, 1630 Chicago Avenue, Suite 1901, Evanston, IL 60201 USA; 6 J.R. Simplot Company, 5369 West Irving Street, Boise, ID 83706 USA; 7 AgBiome Inc, 104 T. W. Alexander Drive, Building 1, Research Triangle Park, NC 27709 USA

## Abstract

**Key message:**

We identified two novel wheat stem rust resistance genes, *Sr-1644-1Sh* and *Sr-1644-5Sh* in *Aegilops sharonensis* that are effective against widely virulent African races of the wheat stem rust pathogen.

**Abstract:**

Stem rust is one of the most important diseases of wheat in the world. When single stem rust resistance (*Sr*) genes are deployed in wheat, they are often rapidly overcome by the pathogen. To this end, we initiated a search for novel sources of resistance in diverse wheat relatives and identified the wild goatgrass species *Aegilops sharonesis* (Sharon goatgrass) as a rich reservoir of resistance to wheat stem rust. The objectives of this study were to discover and map novel *Sr* genes in *Ae. sharonensis* and to explore the possibility of identifying new *Sr* genes by genome-wide association study (GWAS). We developed two biparental populations between resistant and susceptible accessions of *Ae. sharonensis* and performed QTL and linkage analysis. In an F_6_ recombinant inbred line and an F_2_ population, two genes were identified that mapped to the short arm of chromosome 1S^sh^, designated as *Sr-1644-1Sh*, and the long arm of chromosome 5S^sh^, designated as *Sr-1644-5Sh*. The gene *Sr-1644-1Sh* confers a high level of resistance to race TTKSK (a member of the Ug99 race group), while the gene *Sr-1644-5Sh* conditions strong resistance to TRTTF, another widely virulent race found in Yemen. Additionally, GWAS was conducted on 125 diverse *Ae. sharonensis* accessions for stem rust resistance. The gene *Sr-1644-1Sh* was detected by GWAS, while *Sr-1644-5Sh* was not detected, indicating that the effectiveness of GWAS might be affected by marker density, population structure, low allele frequency and other factors.

**Electronic supplementary material:**

The online version of this article (doi:10.1007/s00122-017-2882-8) contains supplementary material, which is available to authorized users.

## Introduction

Stem rust, caused by *Puccinia graminis* f. sp. *tritici* (*Pgt*), has historically been one of the most devastating wheat diseases in many production growing regions in the world. Since the 1950s, stem rust has been effectively controlled mainly through the deployment of cultivars carrying multiple stem rust resistance (*Sr*) genes and elimination of the alternate host, the common barberry bush (*Berberis vulgaris* L.), in North America and Western Europe. However, emergence of the *Pgt* race TTKSK (a member of the Ug99 race group) in east Africa in the 1998–1999 growing season and its spread to other areas has posed a serious threat to world wheat production because of its virulence for several widely used *Sr* genes (Singh et al. 2011). To date, 11 races in the Ug99 race group have been identified and characterised by their diverse virulence spectra on defined *Sr* genes (Newcomb et al. 2016; Singh et al. 2015). Many of these *Sr* genes have been overcome by one or more of these races, including *Sr24* (Jin et al. 2008), *Sr31* (Pretorius et al. 2000), *Sr36* (Jin et al. 2009) and *SrTmp* (Newcomb et al. 2016). Recently, a severe, localized stem rust epidemic developed on the variety Digalu in southern Ethiopia during November–December 2013. Digalu carries *SrTmp* and is resistant to race TTKSK; however, the epidemic was caused by race TKTTF, which is not part of the Ug99 race group (Olivera et al. 2015). Deployment of single *Sr* genes in wheat often leads to the rapid appearance of resistance-breaking strains of *Pgt*. However, the simultaneous deployment of multiple, broad spectrum *Sr* genes in a single cultivar might provide more long term resistance (Singh et al. 2006, 2011).

According to the online *Sr* gene catalogue for wheat, more than 50 *Sr* genes have been identified to date, including tetraploid and hexaploid wheat, and its wild relatives (McIntosh et al. 2013). Most of the genes identified in domesticated wheat have not proven effective against newly emerged *Pgt* races (Singh et al. 2006, 2011). In some cases, this may be due to these *Sr* genes being present in common varieties (Roelfs and McVey 1979) that have been grown on a large scale for a long period of time (e.g. *Sr6*; Tsilo et al. 2009) in areas where stem rust is prevalent, thus exerting extreme pressure on the pathogen to overcome these genes. Wild wheat relatives are a particulary rich source of *Sr* genes, e.g. *Sr24, Sr25, Sr26, Sr32, Sr33, Sr36, Sr37, Sr38, Sr39, Sr40, Sr43, Sr44, Sr45, Sr47, Sr50, Sr51, Sr52*, and *Sr53* which have all been introgressed into hexaploid bread wheat (Bariana and McIntosh 1993; Dundas et al. 2007; Anugrahwati et al. 2008; Liu et al. 2011a, b, 2013; Niu et al. 2011, 2014; Qi et al. 2011; Klindworth et al. 2012; Mago et al. 2013; Periyannan et al. 2013; McIntosh et al. 2013). Many of these genes have already been deployed in cultivated wheat or have entered into breeding programmes and thus may come under a higher risk of being overcome. It is necessary therefore to continue identifying new *Sr* genes and ideally deploy these as multi-*Sr* gene combinations to promote their longevity in the field.

*Aegilops sharonensis* Eig, or Sharon goatgrass (S^sh^ genome), is a wild diploid relative of hexaploid wheat. Some preliminary work on this species (Olivera et al. 2007, 2010, 2013; Bouyioukos et al. 2013; Scott et al. 2014) has demonstrated its potential as a source of new resistance genes to important fungal pathogens (Olivera et al. 2007; Scott et al. 2014). Olivera et al. (2007) reported that the frequency of resistance in *Ae. sharonensis* accessions was highest to wheat powdery mildew (79–83%) and wheat leaf rust (60–77%). Resistance to stem rust was also common, although the percentage of resistant accessions varied markedly depending on the pathogen race—13% to race TTTTF, 69% to TTKSK, and 72% to race QCCJB. The frequency of resistance was intermediate to stripe rust (45%) and low to tan spot (15–29%) and spot blotch (0–34%). Interspecies hybridization between wheat and *Ae. sharonensis* is restricted due to the presence of gametocidal genes in *Ae. sharonensis* (Millet et al. 2014; Knight et al. 2015). Therefore, the introgression of *Ae. sharonensis* genes into wheat has been limited (Millet et al. 2014). For this reason, this species remains a largely untapped reservoir of disease resistance genes. The objectives of this study were to identify novel *Sr* genes in *Ae. sharonensis* and to exploit the possibility of finding *Sr* genes by genome-wide association study (GWAS).

## Materials and methods

### Plant materials

We developed two biparental populations of *Ae. sharonensis*: a recombinant inbred line (RIL_5:6_) and an F_2_ population. The RIL population was developed from a cross between the accessions AEG-2189 (AEG is the first three letters of the word “Aegilops”) and AEG-1644 (Supplementary Table 1), while the F_2_ population was developed from a cross between AEG-2189 and AEG-409 (accessions 2189, 1644 and 409 hereafter). Accessions 1644 and 409 are stem rust resistant parents, while 2189 is a susceptible parent. For both the 2189 × 1644 RIL and the 2189 × 409 F_2_ populations, we phenotyped and genotyped 92 lines/individuals along with the respective parents and two F_1_ plants. The 92 F_6_ lines and 92 F_2_ individuals were randomly selected. For GWAS, we evaluated a diversity panel comprising 125 accessions spanning the geographical range of the species (Supplementary Table 2). Each accession was increased at least twice under controlled glasshouse conditions whereby individual spikes were bagged to prevent out-crossing to reduce heterozygosity prior to genotyping and phenotyping. Spikes of all accessions were bagged to prevent possible out-crossing. Accessions can be obtained from John Innes Centre Germplasm Resources Unit (http://www.jic.ac.uk/germplasm/) and the Harold and Adele Lieberman Germplasm Bank Tel-Aviv University (http://www.genesys-pgr.org/wiews/ISR003). Wheat accessions Chinese Spring and McNair 701 were used as susceptible controls in the rust phenotyping experiments.

### Pathogen races

Three races of *Pgt* with diverse virulences and geographic origins were used in this study to detect resistance genes in *Ae. sharonensis*. Race TPMKC was the predominant race in the United States during the 1990s, and it is virulent on *Sr36* (McVey et al. 2002). Race TRTTF has been identified in Yemen and Ethiopia, and it is virulent to four *Sr* genes that are effective to race TTKSK (*Sr13, Sr36, SrTmp*, and *Sr1RS* ^*Amigo*^) (Olivera et al. 2012). TTKSK was the first race identified in the Ug99 race group and is virulent for *Sr31*, one of the most widely used stem rust resistance genes in wheat (Pretorius et al. 2000).

### Stem rust resistance evaluation

Stem rust phenotyping was conducted in the Biosafety Level-3 Containment Facility on the St. Paul campus of the University of Minnesota. Evaluations were done at the seedling stage, when the second leaves of plants were fully expanded, about 10–12 days after sowing (Williams et al. 1992). The RIL population was tested against races TPMKC, TRTTF, and TTKSK in two replicate experiments with an additional test on selected lines. The 2189 × 409 F_2_ population was tested with race TTKSK. The 125 accessions in the diversity panel were evaluated against races TPMKC, TRTTF, and TTKSK in two replicate experiments with an additional test on selected lines. Protocols for rust inoculations and conditions for subsequent incubation of plants were as described by Scott et al. (2014).

Seeds were sown in super-cell cones (Stuewe and Sons, Inc., Corvallis, OR) or pots filled with 1:1 mixture of steam-sterilized native soil and Sunshine MVP growing mix (Sun Gro Horticulture Distribution Inc., Bellevue, WA) (Scotts-Sierra Horticultural Products Company, Marysville, OH). Five seeds per replicate per line were sown in the pot for 2189 × 1644 RIL population and the association panel, while 2189 × 409 F_2_ individuals were sown in cones. Water soluble 15-0-15 fertilizer (JR Peters Dark Weather) was applied when the plants emerged and then 20-10-20 fertilizer was applied (JR Peters 20-10-20 Peat-Lite) 1 and 7 days after inoculation. The seedlings were grown in the greenhouse at 20–23 °C with 16/8 h (day/night) photoperiod. At the second leaf stage, 10–12 days after sowing, when the second leaf was fully expanded, the seedlings were inoculated with *Pgt* urediniospores (Williams et al. 1992).

Infection types were recorded for each plant at 12–14 days post-inoculation, using the infection type (IT) scale described by Stakman et al. (1962), where 0 = immune, necrotic flecks, 1 = minute uredinia surrounded by distinct necrosis, 2 = small to medium sized uredinia surrounded by chlorosis or necrosis, 3 = medium sized uredinia sometimes surrounded by chlorosis, and 4 = large uredinia without chlorosis. The additional notations of “−” or “+” for ITs 1, 2, or 3 were applied when the uredinia were slightly smaller or larger than those classically described. For the purpose of mapping, only the predominant (first listed) IT was used. ITs 0, 1−, 1, 1+, 2−, 2, 2+, 3−, 3, 3+, and 4 were converted into the respective numeric values 0, 0.33, 0.67, 1, 1.33, 1.67, 2, 2.33, 2.67, 3, 3.33, and 4 to make the scores amenable to quantitative genetics. On average, 4.0 plants for 2189 × 1644 RIL and 4.1 plants for the association panel were scored, while the 2189 × 409 F_2_ plants were scored as individuals.

### Oligo pool assay (OPA) genotyping

We used the whole genome shotgun (WGS) assembly of *Ae. sharonensis* accession 1644 (Mayer et al. 2014) as a reference for SNP calling. We also selected accession 2189 for WGS assembly because of its susceptibility to race TTKSK and its use as a parent in the two mapping populaions. WGS reads from the *Ae. sharonensis* accession 2189 (PRJEB5333) were mapped to the 1644 reference using Burrows-Wheeler Aligner (Li and Durbin 2009) and SNPs were called using samtools mpileup (Li et al. 2009). SNPs were filtered based on unambiguous read support. We also mapped reads from the leaf transcriptomes of 16 accessions (Supplementary Table 3) (PRJEB5340) to the reference using TopHat (Kim et al. 2013) and called SNPs between either of the three accessions 409, 2020 and 2172 versus accession 2189. SNPs were filtered based on unambiguous read support. To comply with OPA genotyping requirements, all positions with SNPs from either genomic or transcriptomic analysis were then filtered for having no additional variation called within 80 bp in both directions and containing contiguous flanking sequence of ≥100 bp in both directions. Contigs of the reference assembly were anchored to the barley (*Hordeum vulgare* L.) genome (Mayer et al. 2014) by synteny. This was done by best reciprocal BLAST hit to barley proteins. For each cM position in the barley genome, we selected up to three of our SNPs that were located on anchored contigs. Additionally, we selected randomly chosen SNPs from our list. In this way, 3072 SNPs were selected and sent to the University of California, Los Angeles, Neuroscience Genomics Core (UNGC) for usability scoring. According to this score, we selected a final set of 1536 SNPs as probes for our OPA assay (Supplementary Table 4). All SNPs selected had an Illumina final score of 0.604 or higher.

For the 2189 × 409 F_2_ population, we extracted DNA from each F_2_ plant. For the 2189 × 1644 RIL population and diversity panel, each line was planted in a single pot. Leaves were taken from a single young plant and DNA was extracted following the protocol described by Yu et al. (2010). OPA genotyping was done at the UNGC and the genotypes were manually curated.

### DNA and RNA extraction for sequencing

Genomic DNA was isolated from purified nuclei (Brenchley et al. 2012) and sequenced by The Genome Analysis Centre (TGAC; http://www.tgac.ac.uk, UK) and Eurofins (http://www.eurofins.eu, Ebersberg, Germany) on an Illumina HiSeq2000. Accession 1644 was sequenced from three paired-end (PE) libraries with an insert size of 300 bp (International Wheat Genome Sequencing Consortium 2014). Accession 2189 was sequenced from one overlapping 200 bp PE library, two 300 bp PE libraries, one 700 bp PE library, and two mate-pair-like libraries using a proprietary protocol (Eurofins, Ebersberg, Germany) with 3000 and 8000 bp insert sizes. Raw data have been made available through the European Nucleotide Archive (ENA), Study IDs PRJEB4849 and PRJEB5333.

Total RNA was extracted from tissue obtained from second and third leaves of 16 *Ae. sharonensis* accessions using the TRI Reagent (Sigma-Alrich) according to the manufacturer’s instructions (Supplementary Table 3). RNA samples were depleted of DNA by treatment with DNase and further purified using the RNeasy MinElute kit (Qiagen Ltd.). RNA yield and quality were assessed using a Nanodrop spectrophotometer (Thermo Scientific) and a Bioanalyzer 2100 Nanochip (Agilent Technologies). RNA was sequenced as paired-ends at TGAC by multiplexing four samples per lane on an Illumina HiSeq 2000 and using the Illumina TruSeq RNA kit. Raw data have been made available through ENA, Study ID PRJEB5340.

### ANOVA, linkage map construction, and QTL mapping

The program PAST (Belkhir et al. 1996–2004; http://univ-montp2.fr/~genetix) was used for ANOVA. The Map Manager QTX2b program (Manly et al. 2001), with *p* value set to 0.001, was used to make the genetic linkage maps for each population using the Kosambi function (Kosambi 1944). Markers were selected to include those with the least missing data points (<4), and minimum data points for a minor allele (>28 for the RIL population and >8 for the F_2_ population). To take into account the different recombination rates between the F_2_ and F_6_ populations and the different markers successfully mapped in them, a consensus map was manually constructed based on common markers on the individual linkage maps. The common markers’ genetic positions were averaged and the non-common markers’ genetic positions were determined relative to the mean interval. In addition to QTX, Carthagene was also employed to generate genetic linkage maps using the Kosambi function (Kosambi 1944). We used command “Build” at LOD 3 to make individual genetic linkage maps for the 2189 × 1644 RIL and 2189 × 409 F_2_ populations (Supplementary Table 5) and used command “Mergor” and then “Build” to construct the consensus map (Supplementary Table 6). The consensus maps constructed manually and with Carthagene were found to be highly correlated (Supplementary Fig. 1). However, more than 200 markers on the individual linkage maps for the 2189 × 1644 RIL and 2189 × 409 F_2_ populations were left out of the Carthagene consensus map. Therefore, we decided to proceed with the manually constructed consensus map for all subsequent analysis. QTL mapping for biparental populations was conducted using QTL Cartographer (Wang et al. 2006) using the default parameters except a 1 cM step was applied.

For the diversity panel, the heterozygous genotypes were considered as missing. Data were filtered according to the following criteria. First, lines with 10% or more missing data points for all markers were removed. Second, a SNP that had a minor allele frequency of less than 5% was removed. Third, a SNP with missing data points in more than 10% of the lines was removed. Finally, three lines that had a cluster distance (Euclidean-single linkage) of less than 1.5 were removed.

### Linkage disequilibrium

The program Tassel 4.04 (http://www.maizegenetics.net) was used to estimate the linkage disequilibrium (LD) parameter *r* ^2^ among loci. LD was first computed for all unlinked loci (>50 cM and inter-chromosomal) of the whole genome and the pair-wise significance was computed. The parametric 95th percentile was taken as a threshold of *r* ^2^. Chromosome-wide *r* ^2^ was then separately computed for each chromosome on different loci. Then all *r* ^2^ were pooled from the seven chromosomes. The *r* ^2^ was plotted against genetic distance on the whole genome. The LOESS procedure of *r* of PAST (Belkhir et al. 1996–2004; http://univ-montp2.fr/~genetix) was used to draw a smooth line of second-degree LOESS with a smooth factor of 0.5 (Cleveland 1979). The intersection of the LOESS curve and the threshold line was considered as the estimate of the extent of LD in the genome.

### Population structure, principal component, and kinship analysis

Forty markers that had a genetic distance of ~10 cM were selected for population structure analysis. The program Structure 2.3.3 (Pritchard et al. 2000) was used to determine the number of subpopulations with the default admixture model and correlated allele frequencies (Falush et al. 2003). The program was run for each value of the subpopulation number (*K* = 1 to *K* = 6), with burn-in phase of 10^5^ iterations and a sampling phase of 10^5^ replicates. Because the optimal *K* was not able to be determined based on Ln *p* (*X*|*K*), the first and second derivatives of Ln *p* (*X*|*K*) were also computed and Δ*K* was used to determine the optimal *K*. Accessions were discretely assigned to the subpopulation for which the probability was the largest among the subpopulations. The program PAST (Belkhir et al. 1996–2004; http://univ-montp2.fr/~genetix) was used to conduct the principal component analysis (PCA). For kinship analysis, SPAGeDi 1.3a (Hardy and Vekemans 2002) was used to compute the relative kinship matrix using 388 markers.

### Marker-stem rust resistance association analysis

Tassel 4.04 was also used for marker-resistance association analysis. Associations between markers and transformed stem rust resistance scores were tested using a general linear model (GLM) and also a compressed mixed-effects linear model (MLM) where the marker being tested was considered as a fixed-effect factor, population structure factors as covariates, and kinship as a random-effect factor (Yu et al. 2006). Significance of associations between markers and stem rust resistance was based on 1000 permutations (Churchill and Doerge 1994) with GLM and MLM. Tassel does not allow experiment-wise permutations; therefore, permutation tests were performed by shuffling the phenotypic data 1000 times using a custom Python script, running the shuffled data in Tassel, and sorting *p* values of each test in an Excel spreadsheet to get the smallest *p* value of each test. The −log_10_ *p* at the 95th percentile of the 1000 permutations was taken as the threshold.

Removal of closely related accessions to generate a trained population was based on cluster analysis with 40 markers using program PAST (Belkhir et al. 1996–2004; http://univ-montp2.fr/~genetix). We removed 23 accessions with less than 0.4 of the relative distance (branch distance of an accession/the longest distance, Euclidean-single linkage). Each of the 23 accessions was randomly selected from a pair of the closest ones in the cluster.

## Results

### Phenotyping and ANOVA

**Fig. 1:**
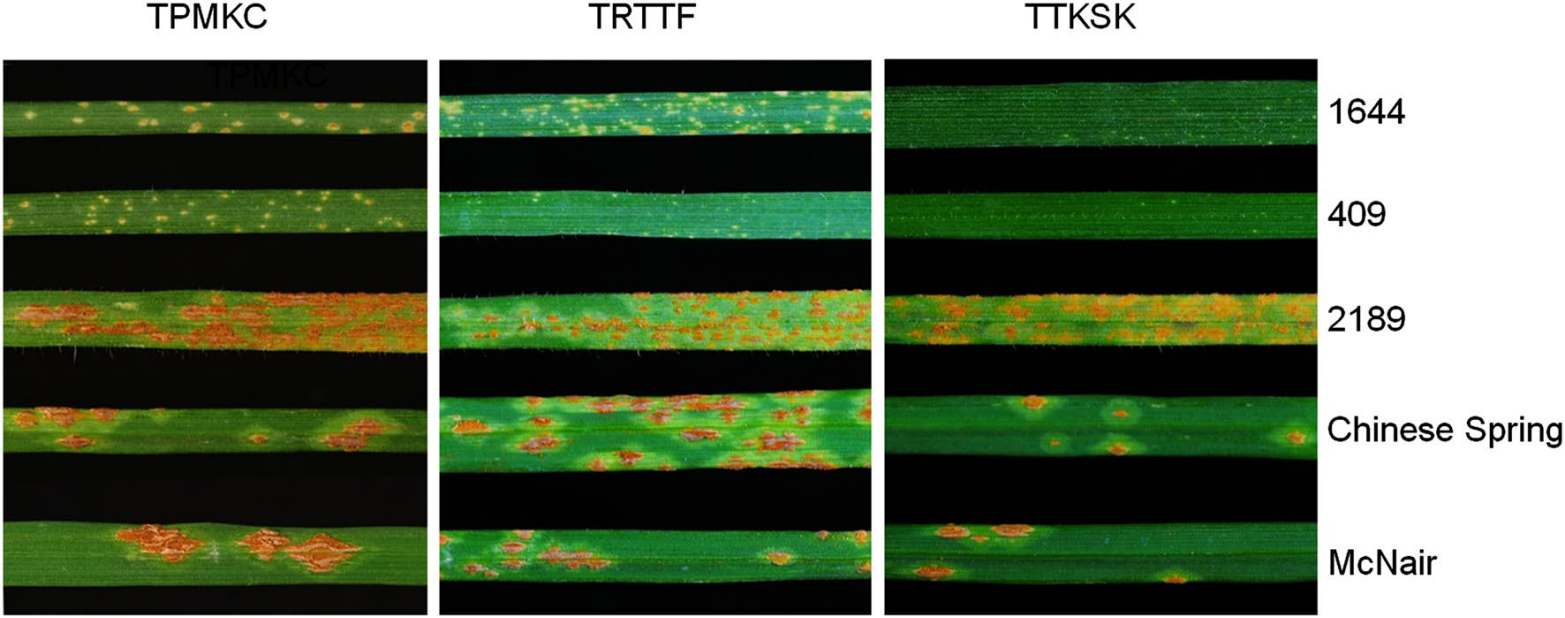
Evaluation of *Ae. sharonensis* parental accessions and wheat controls for stem rust resistance at the seedling stage. Three *Ae. sharonensis* parental accessions (1644, 409, 2189) and two wheat controls (Chinese Spring and McNair) were tested against *Pgt* races TPMKC, TRTTF, and TTKSK

The ITs for the parental accessions 1644, 409 and 2189 are shown in Fig. 1. Accessions 1644 and 409, showed high levels of resistance (ranging between 0; and 1;) to all three races of TPMKC (USA), TRTTF (Yemen), and TTKSK (Ug99; Kenya). In contrast, accession 2189 was susceptible to all three races (Fig. 1).

**Fig. 2:**
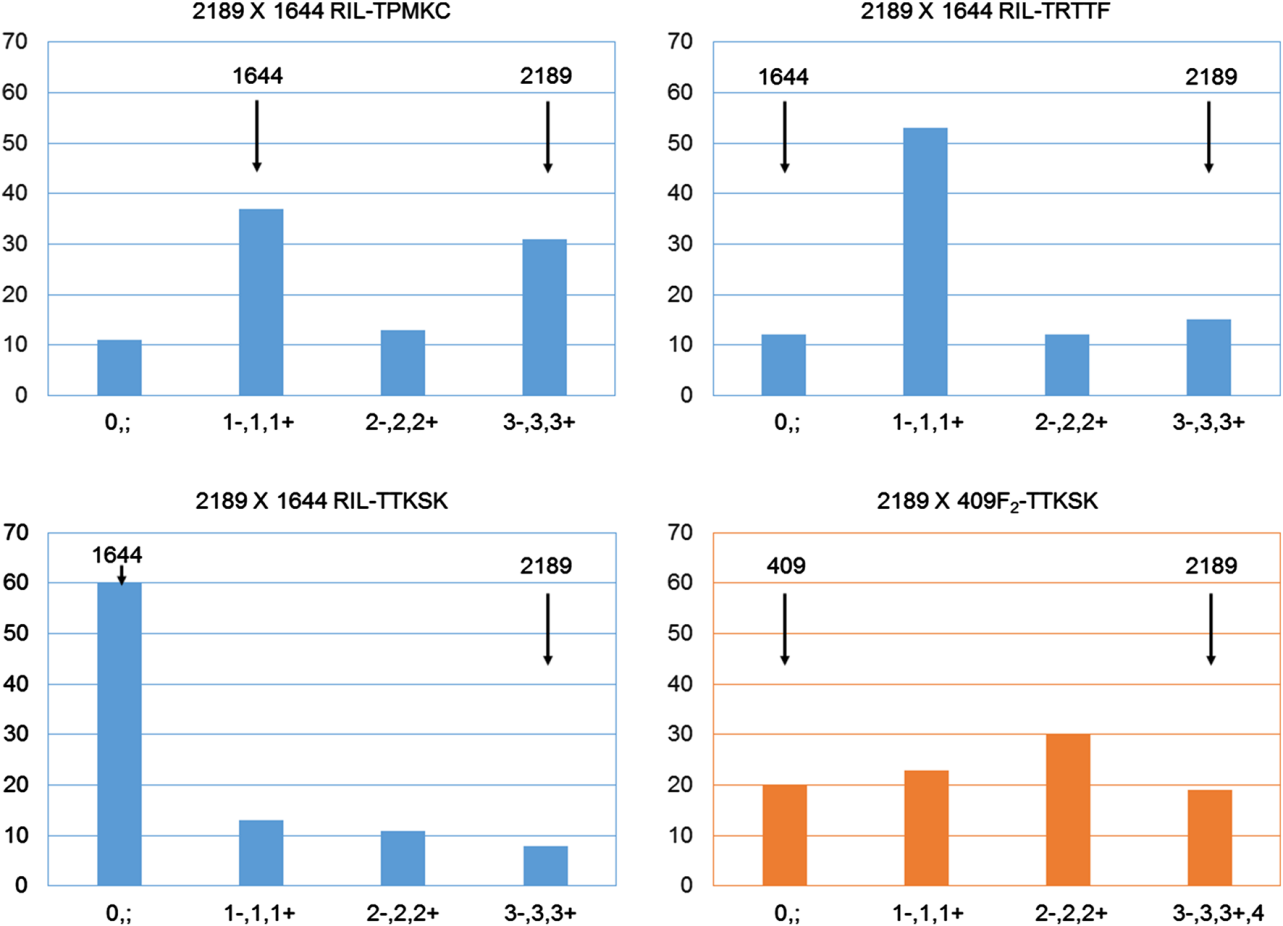
Wheat stem rust infection type distributions in biparental populations of *Ae. sharonensis*. The *X*-axis indicates the infection type while the *Y*-axis indicates the number of individual lines/plant. Ninety-two 2189 × 1644 RILs (*blue*) were scored for their reactions to *Pgt* races TPMKC, TRTTF and TTKSK, while 92 2189 × 409 F_2_ plants (*orange*) were scored for their reaction to race TTKSK only. Parental phenotypes are indicated by *vertical arrows*. (Color figure online)

In the biparental populations made between the resistant and susceptible accessions, segregation for resistance was observed with all three races (Fig. 2). Plants showing IT 0; 1, or 2 were considered resistant, while the ones showing 3 or 4 were considered susceptible. In the 2189 × 1644 RIL population, the ratio of the number of resistant to susceptible lines was very different from 1:1. For example, to race TTKSK, the number of resistant lines (*n* = 76) vastly exceeded the number of susceptible lines (*n* = 8) or segregating lines (*n* = 8) (i.e. showing both resistant and susceptible plants within the same line; Supplementary Table 1). In the 2189 × 409 F_2_ population, the ratio of the number of resistant to susceptible plants was close to 3:1 (*p* = 0.25) indicative of a single dominant resistance gene in 409 effective against TTKSK.

ANOVA was performed for the 2189 × 1644 RIL population and the association panel on two replicates (for QTL and association mapping, all scored plants were included). Differences among the RILs were highly significant based on the *F* test (Supplimentary Table 7). For the association mapping panel comprised of 125 accessions, highly significant differences were found for the IT scores to all three races (Supplementary Table 8).

### Genetic linkage map

The genetic linkage map for the 2189 × 1644 RIL population was created based on a RIL population model, while the genetic linkage map for the 2189 × 409 F_2_ population was based on an F_2_ population model. Since the RIL and F_2_ are different types of populations, a consensus linkage map was manually constructed based on the two individual maps. Based on the collinearity between *Ae. sharonensis* and barley, we were able to assign the individual chromosome groups and to determine the orientation of each chromosome. We assigned chromosome names 1S^Sh^ to 7S^Sh^ corresponding to 1H–7H (Supplementary Fig. 2).

The consensus genetic linkage map is composed of 727 OPA markers. The total length of the seven chromosomes is 630.7 cM with an average distance of 0.9 cM between two neighbouring markers. The largest gap in the map is 18.2 cM on chromosome 2S^Sh^. Two other big gaps (>10 cM) are located on the long arm of chromosome 4S^Sh^ and the short arm of chromosome 5S^Sh^. While 4S^Sh^ is the shortest chromosome (58.8 cM), 5S^Sh^ is the longest one (109.7 cM).

### Biparental population mapping

**Fig. 3:**
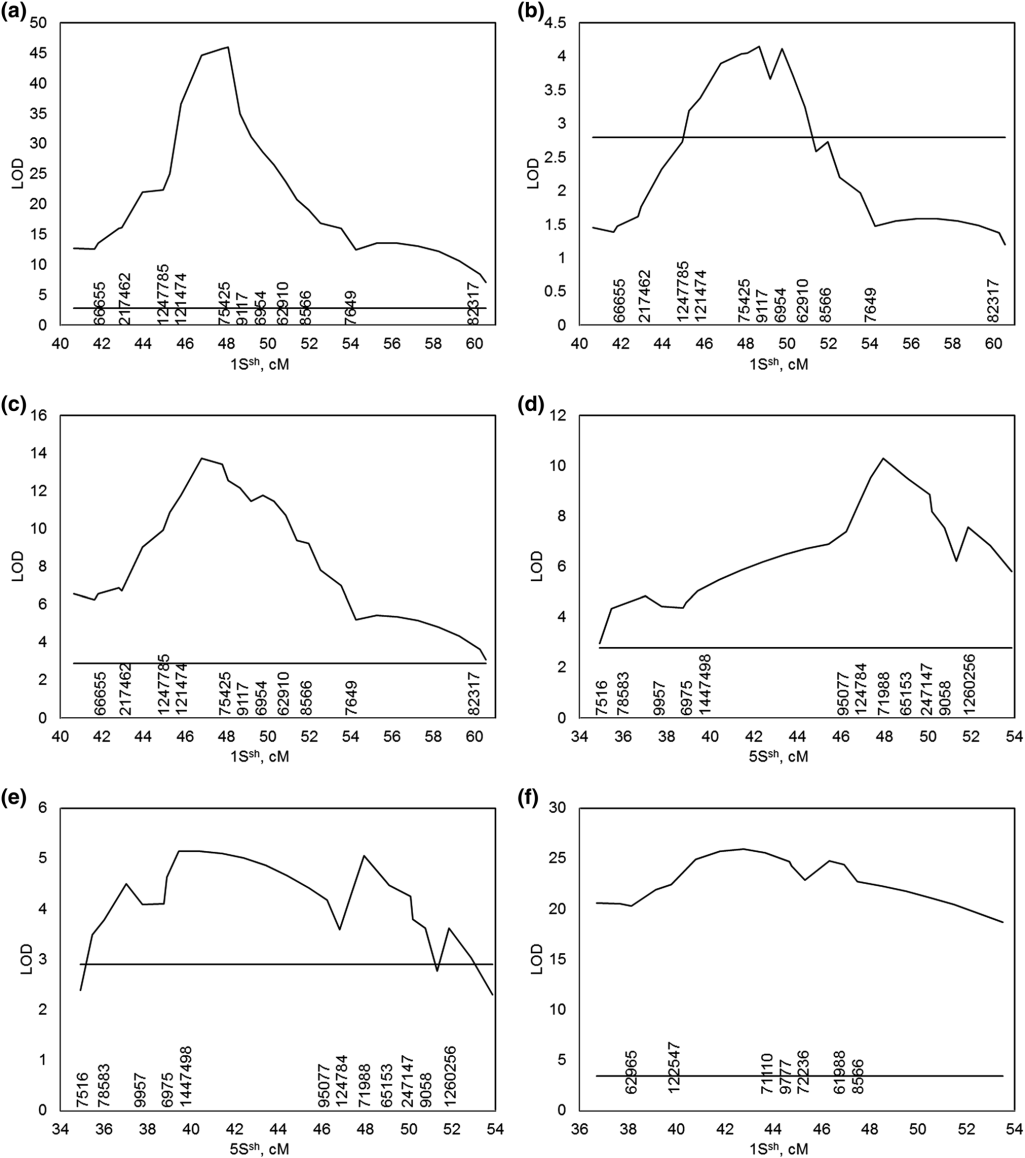
QTL interval mapping in the 2189 × 1644 RIL and 2189 × 409 F_2_ populations were performed on the stem rust resistance to pathogen races TPMKC, TRTTF, and TTKSK. The *horizontal lines* indicate the thresholds based on 1000 permutations. **a** Scan on partial chromosome 1S^sh^ for 2189 × 1644 RIL against TPMKC, **b** scan on partial chromosome 1S^sh^ for 2189 × 1644 RIL against TRTTF, **c** scan on partial chromosome 1S^sh^ for 2189 × 1644 RIL against TTKSK, **d** scan on partial chromosome 5S^sh^ for 2189 × 1644 RIL against TRTTF, **e** scan on partial chromosome 5S^sh^ for 2189 × 1644 RIL against TTKSK, **f** scan on partial chromosome 1S^sh^ for 2189 × 409 F_2_ against TTKSK

QTL mapping was used to identify the genetic location of genes conferring stem rust resistance in the two biparental genetic populations. The QTL mapping was done using the genetic linkage maps generated for the F_2_ and RIL populations, respectively. We used 1000 permutations to determine the LOD threshold for the significance of a QTL. The significant QTLs are listed in Table 1. Two QTLs with large phenotypic effects were identified and mapped to chromosome 1S^Sh^ and 5S^Sh^ in both mapping populations. Although the map positions for the two QTLs peak in the populations at slightly different positions, they were located in the same narrow region (Fig. 3). Thus, we tentatively named them Sr-QTL-1Sh and Sr-QTL-5Sh, respectively.

**Table 1:** Significant QTL for stem rust resistance in biparental populations (2189 × 1644 recombinant inbred line and 2189 × 409 F_2_ populations) of *Aegilops sharonensis* to races TPMKC, TRTTF, and TTKSK identified by interval mapping

Population	Race	Chr	cM	CMP	LOD	*R* ^2^ (%)	*A*
2189 × 1644 RIL	TPMKC	1S^Sh^	54.5	75,425	46.0	89	−1.06
	TRTTF	1S^Sh^	54.8	9117	4.2	19	−0.37
	TRTTF	5S^Sh^	49.6	71,988	10.3	40	−0.54
	TTKSK	1S^Sh^	52.8	121,474	13.7	51	−0.68
	TTKSK	5S^Sh^	41.6	1,447,498	5.2	23	−0.46
2189 × 409 F_2_	TTKSK	1S^Sh^	52.7	71,110	26.0	82	−1.15

Threshold: LOD 2.9, 2.8, and 2.9 for RIL at *p* = 0.05 against TPMKC, TRTTF, and TTKSK, respectively and for F_2_ population threshold: LOD 3.4 at *p* = 0.05 based on 1000 permutation

*Chr* chromosome, *CMP* the closet marker, *R* ^2^ explained phenotypic variation in percentage, *A* additive effect

The two QTL behaved differently in the two populations against the three races (Fig. 3). In the 2189 × 1644 RIL population, the Sr-QTL-1Sh conferred a high level of resistance to race TTKSK (explaining 51% of the phenotypic variation) and an extremely high level of resistance to TPMKC (explaining 89% of the variation). However, it conferred a lower level of resistance to race TRTTF (explaining only 19% of the variation). In contrast, Sr-QTL-5Sh conferred a high level of resistance to TRTTF (explaining 40% variation; Table 1). In the 2189 × 409 F_2_ population, the Sr-QTL-1Sh conferred a high level of resistance to TTKSK (explaining 82% of the variation), but the Sr-QTL-5Sh did not confer resistance to TTKSK (Table 1).

### Linkage disequilibrium

**Fig. 4:**
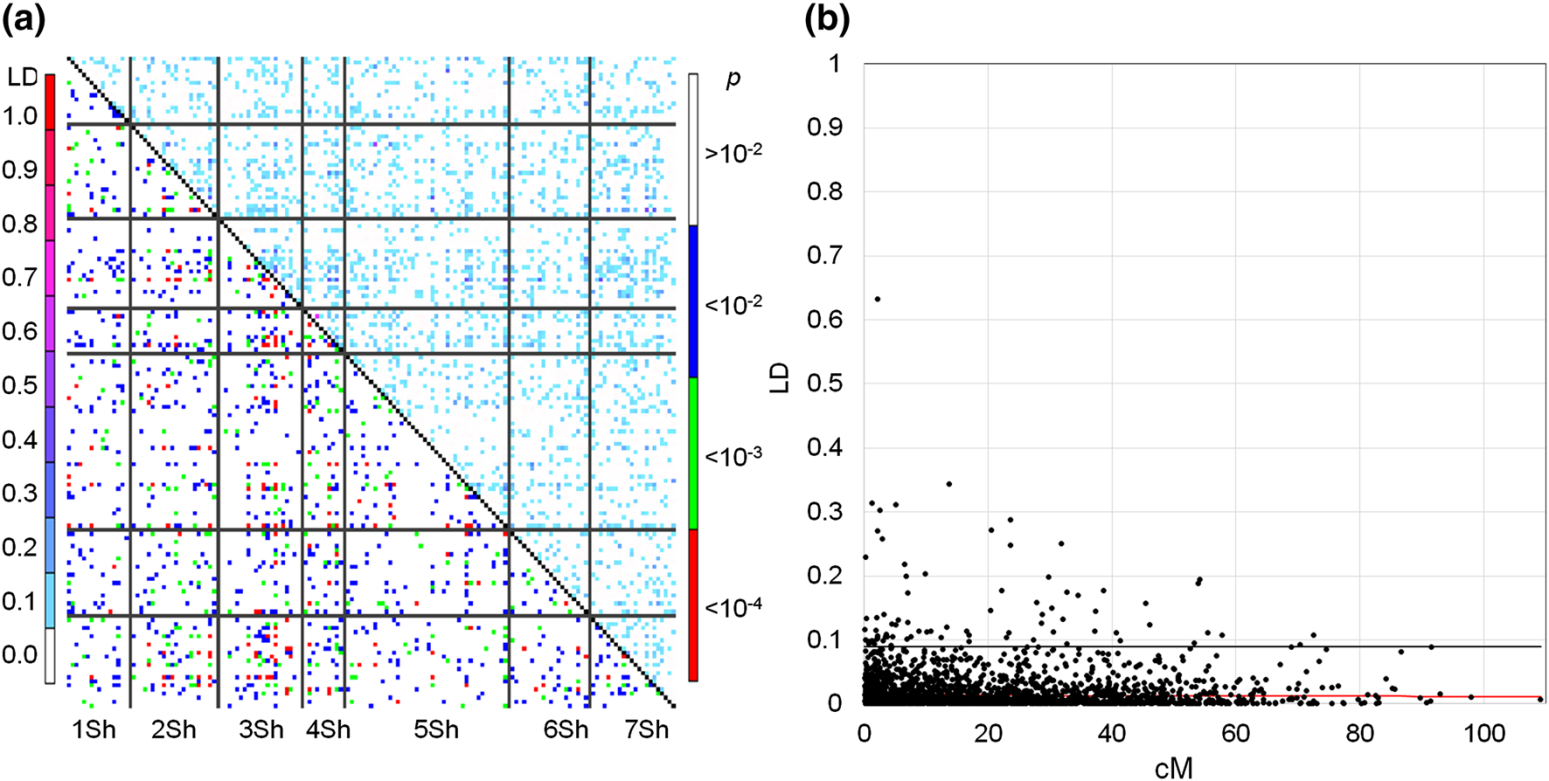
LD (*r* ^2^) and genome-wide average LD decay estimated from 125 *Ae. sharonensis* accessions. **a** The *lower triangle* represents the linkage disequilibrium (LD) while the *upper triangle* represents the *p* values for the LD. In **b**, the *horizontal line* indicates the threshold and the *red line represents* the LOESS curve. (Color figure online)

Among the 727 OPA markers comprising the consensus genetic linkage map, 153 non-redundant markers (only one marker at a genetic linkage position) were selected for linkage disequilibrium analysis. The *r* ^2^ results and respective *p* values are shown in Fig. 4. Based on whole genome unlinked (>50 cM or inter-chromosomal) markers, the significance threshold for LD was determined to be 0.09. Since LOESS curves did not intercept the threshold line (Fig. 4), the extent of LD for the whole genome could not be determined.

### Population structure

**Fig. 5:**
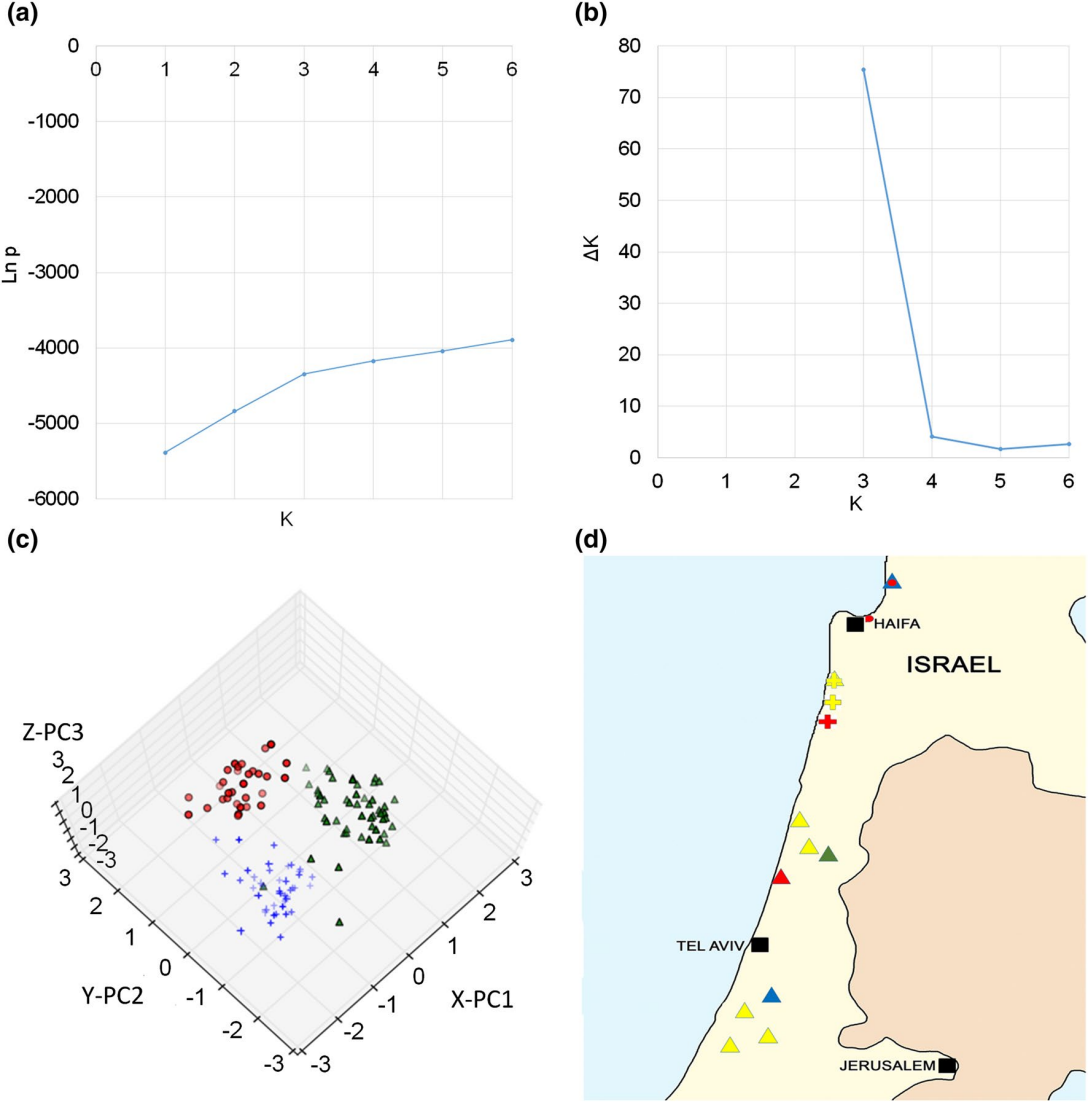
Population structure analysis of 125 *Ae. sharonensis* accessions. **a** Ln *p* and Delta *K* plot over subpopulation number *K*. **b** Delta *K* plot over subpopulation number *K*. **c** Projections of the PCA clouds in the space defined by the first three principal components. *Three different shapes* indicate the subpopulation: *red circle* subpopulation 1; *blue cross* subpopulation 2; *green triangle* subpopulation 3. **d** Geographical distribution of three subpopulations with various levels of resistance to TTKSK. The *shape* indicates the subpopulation: *circle* subpopulation 1; *cross* subpopulation 2; *triangle* subpopulation 3. The *color* indicates the infection type (IT): *green* 0, *blue* 1–11+, *yellow* 2–22+, *red* 3–33+. (Color figure online)

The posterior probability of the data did not peak in the range of 1–6 subpopulations. However, the Δ*K* of the Ln *p* (*X*|*N*) on 30 replicates had the highest value at the three subpopulations (Fig. 5a, b). Therefore, three subpopulations were identified based on the Δ*K* method proposed by Evanno et al. (2005). The three subpopulations included 31, 42, and 52 lines, which had a mean *F*_ST_ equal to 0.34, 0.30, and 0.31, respectively.

The principal component analysis (PCA) confirmed the identification of the three subpopulations by the program PAST. Figure 5c shows projections of the PCA clouds in the space defined by three orthogonal axes (the first three components). Three clouds were formed in the space although four accessions (AS_06480, AS_06485, AS_06489, and AS_06637) of subpopulation 3 fell into subpopulation 1.

In general, the *Ae. sharonensis* accessions of subpopulation 2 were more resistant than the ones of subpopulation 1, and accessions of subpopulation 3 were less resistant than the ones of subpopulation 2 against races TPMKC, TRTTF, and TTKSK. Within subpopulation 1, only a few of the accessions showed resistance to TPMKC. Almost half of the accessions were resistant to TRTTF, and the majority of the accessions were resistant to TTKSK. Within subpopulation 2, only 1 out of the 42 accessions was susceptible to TRTTF or TTKSK, but 13 accessions were susceptible to TPMKC. Within subpopulation 3, while the vast majority of the 42 accessions were resistant to TRTTF or TTKSK, two-fifths of the accessions were susceptible to TPMKC (Supplementary Table 2).

Geographically, subpopulation 1 (characterized by susceptibility, in particular to race TTKSK) was found in an area north of Haifa (Akko Plain region) (Fig. 5d). In contrast, subpopulation 2 was collected south of, but close to Haifa (Coast of Carmel region), and all the accessions, but one, were resistant to TTKSK. Subpopulation 3 was collected mainly around Tel Aviv (Sharon Plain and Phillistean Plain regions), but included some accessions from the Haifa area. The majority of accessions from this subpopulation were resistant to TTKSK.

### Association mapping

**Fig. 6:**
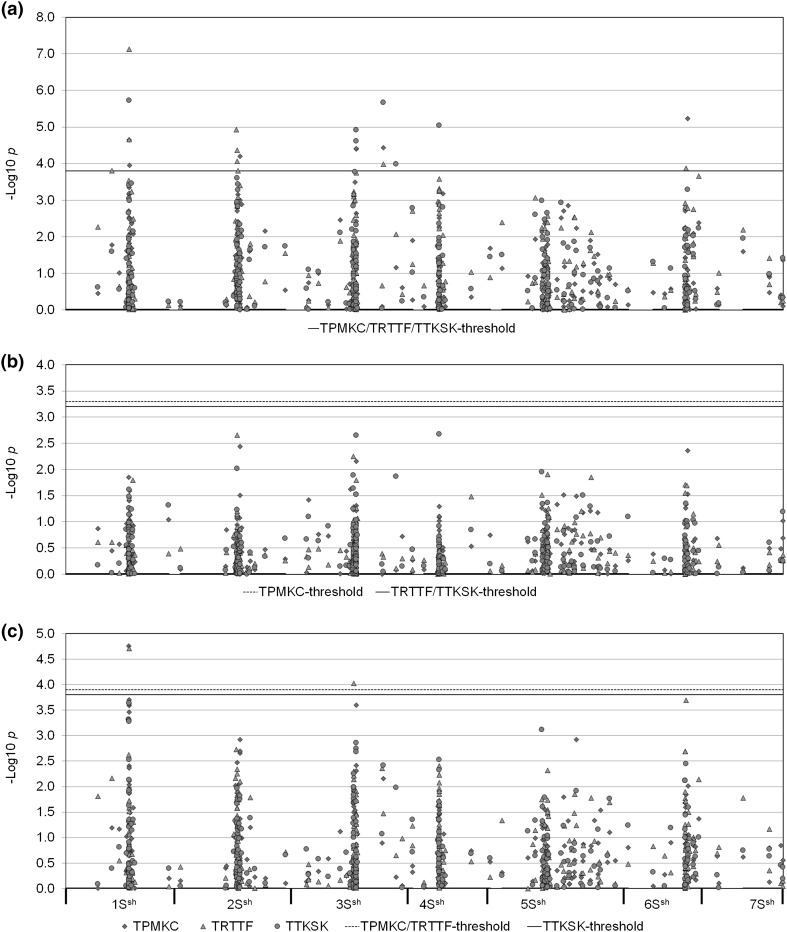
Genome-wide association study of stem rust resistance in *Ae. sharonensis*. GLM and MLM analyses were performed on 421 markers and the three *Pgt* races TPMKC, TRTTF, and TTKSK were used. *Vertical axes* represents −log10 *p* values of the *p* value of the association. **a** GLM on 125 accessions; **b** MLM on 125 accessions; **c** GLM-TP on 102 accessions

The results of marker-resistance association analysis are shown in Fig. 6. Based on GLM, seven markers, 7283 and 6954 on 1S^Sh^, 91,247 on 2S^Sh^, 70,039, 61,971, and 71,850 on 3S^Sh^, and 60,772 on 6S^Sh^ were significantly associated with resistance to TPMKC at the −log_10_ *p* = 3.9. These markers explained 12–16% of the phenotypic variation of the resistance to TPMKC. For resistance to TRTTF, eight markers, 7283 and 7215 on 1S^Sh^, 62,411, 103,660 and 246,912 on 2S^Sh^, 71,850 on 3S^Sh^, 62,473 on 6S^sh^ and 1,248,445 on 7S^Sh^ were significantly associated with resistance. These markers explained 11–22% of the phenotypic variation. Against TTKSK, six markers, 7283 on 1S^Sh^, 71,850, 70,039, 61,971, and 70,177 on 3S^Sh^, and 7148 on 4S^Sh^, were significantly associated with resistance. They explained 12–18% of the phenotypic variation of the resistance.

Based on a mixed linear model (MLM), three markers, 91,247 on 2S^Sh^, 61,971 on 3S^Sh^, and 60,772 on 6S^Sh^ were significantly associated with resistance to TPMKC at the arbitrary threshold −log_10_ *p* = 2. These markers explained 7, 6, and 7% of the phenotypic variation for resistance to TPMKC, respectively. For resistance to TRTTF, two markers, namely 103,660 on 2S^Sh^ and 63,580 on 3S^Sh^ were found significantly associated with resistance, explaining 8 and 6% of the phenotypic variation, respectively. Three markers, 103,660 on 2S^sh^, 61,971 on 3S^Sh^, and 7148 on 4S^Sh^, were significantly associated with resistance to TTKSK. They explained 6, 8, and 8% of the phenotypic variation for resistance, respectively. However, none of these marker-resistance associations reached the threshold based on 1000 permutations (Fig. 6b).

Due to possible over-correcting for population structure and kinship (Pasam et al. 2012; Zegeye et al. 2014), we developed a different GLM approach, which does not involve statistical correction for these factors. To this end, we trained the population by manually removing 23 accessions which we found to be closely related to another. With GLM on the trained population (GLM-TP; Fig. 6c), one marker, 62,024 on 1S^Sh^, was significantly associated with resistance to TPMKC at the −log_10_ *p* = 4.0. This marker explained 17% of the phenotypic variation for resistance to this race. For resistance to TRTTF, two markers, namely 7283 on 1S^Sh^ and 9115 on 3S^Sh^ were found significantly associated with resistance. The two markers explained 17 and 14% of the phenotypic variation, respectively. Against TTKSK, no marker was found significantly associated with resistance. The markers found on chromosome 1S^Sh^ are close to Sr-QTL-1Sh: marker 62,024 mapped to the same position (54.5 cM, 1S^Sh^) as marker 75,425, which is the marker closest to the QTL peak against TPMKC, while marker 7283 mapped to the same position (54.8 cM, 1S^Sh^) as marker 9117, which is the marker closest to the QTL peak against TRTTF in the 2189 × 1644 RIL population (Fig. 3; Table 1).

## Discussion

### Consensus genetic linkage map

*Aegilops sharonensis* is a wild diploid wheat relative native to Israel and southern Lebanon. The limited genetic and molecular work on this species (Olivera et al. 2007, 2010, 2013; Bouyioukos et al. 2013; Scott et al. 2014) includes the recent construction of a genetic linkage map based on Diversity Technology Array (DArT) markers and simple sequence repeats (SSR) (Olivera et al. 2013). However, some of the linkage groups were incomplete and also characterized by large gaps between markers. In this study, we designed 1536 OPA markers based on the collinearity between *Ae. sharonensis* and barley using cDNA and genomic shot-gun sequences from key *Ae. sharonensis* accessions.

OPA has been applied in many molecular genetic studies of wheat and barley due to its high quality and throughput (Akhunov et al. 2009; Close et al. 2009; Muñoz-Amatriaín et al. 2011). In this study, we developed a consensus OPA genetic linkage map based on two different types of biparental populations, namely one RIL and one F_2_ population. This consensus map consists of 727 OPA markers. Although there are three gaps larger than 10 cM, the average distance between two neighbouring markers was 0.9 cM. There is a high level of collinearity between the barley and *Ae. sharonensis* chromosomes. Based on BLAST of the mapped *Ae. sharonensis* OPA marker sequences against barley genome sequences, the seven *Ae. sharonensis* chromosomes appear to be well covered with markers, except the short arms of chromosomes 3S^Sh^ and 7S^Sh^.

### Biparental population mapping

We identified two QTL for stem rust resistance to various *Pgt* races including TPMKC, TRTTF, and TTKSK. The two QTL behaved differently in the two populations against these specific races. For example, in the 2189 × 1644 RIL population, *Sr-QTL-1Sh* conferred an extremely high level of resistance to TPMKC but only weak resistance to TRTTF, indicating that the *Sr* gene at this locus confers race-specific resistance. Similarly, in this population, *Sr-QTL-5Sh* conferred a high level of resistance to TRTTF, a relatively low level of resistance to TTKSK, and no significantly detectable resistance to TPMKC (Table 1). Since Sr-QTL-1Sh conferred a high level of resistance, we designated it as gene *Sr-1644-1Sh*, which indicates the gene is from *Ae. sharonensis* accession 1644 and located on chromosome 1S^Sh^. Likewise, we designated the QTL on chromosome 5S^Sh^ as gene *Sr-1644-5Sh*.

So far, no stem rust resistance gene from tetraploid or hexaploid wheat has been mapped to the short arm of chromosome group 1. However, in the wild diploid species *Ae. tauschii*, the D genome progenitor of wheat, two genes *Sr33* and *Sr45* are located on the 1D short arm (Periyannan et al. 2013, 2014). Both *Sr33* and *Sr45* were recently cloned (Periyannan et al. 2013; Steuernagel et al. 2016). Whether the gene *Sr-1644-1Sh* is an ortholog or homoeolog to any of these genes on the short arm of group 1 is presently unknown. In rye, *Sr31* (*Sr1RS* ^*Amigo*^), which has been introgressed into wheat, is located on the short arm of chromosome 1R (Wanyera et al. 2006), while another gene from rye, *Sr50*, maps close to the centromere on the short arm of rye chromosome 1 (Mago et al. 2015).

In wheat, the gene *Sr30* maps to 5DL (Hiebert et al. 2010), but it is not effective against TTKSK. Another gene, *Sr49*, maps to 5BL (Bansal et al. 2015). *Sr53* was recently introgressed from *Aegilops geniculata* to chromosome arm 5DL of wheat (Liu et al. 2011). In barley, the stem rust resistance locus *rpg4*/*Rpg5* is located on the long arm of 5H (Wang et al. 2013). On the *Ae. sharonensis* linkage map, the gene *Sr-1644-5Sh* is close to *Sr30*. The molecular relationship, if any, between the genes *Sr-1644-5Sh, Sr30, Sr49, Sr53* and *rpg4*/*Rpg5* will require further studies.

### Genome-wide association study

Some studies considered the extent of LD as the genetic or physical distance taken for a decay of *r* ^2^ to be an arbitrary value, mostly 0.10 (Remington et al. 2001; Nordborg et al. 2002; Palaisa et al. 2003). We chose the 95th percentile of the distribution of unlinked-*r* ^2^ estimates, including those inter-chromosomal *r* ^2^, as a threshold, which is 0.09 (Fig. 4). The threshold line did not intercept the LOESS regression of the *r* ^2^ across the whole genome, so we were not able to determine the extent of decay. This suggests that the LD of this diversity collection has decayed beyond a statistically detectable level for the marker density in the present map. With decayed LD, the association accuracy of genotype with phenotype (here, disease resistance) should increase while the detecting capability of an association might decrease.

The *Ae. sharonensis* accessions used in this study were collected within Israel. Structure analysis using the program Structure 3 combined with the ad hoc statistic Δ*K* method proposed by Evanno et al. (2005) identified three sub-populations, indicating some degree of diversity in the collection. A similar level of genetic diversity was also reported in another study (Olivera et al. 2009). The PCA defined three subpopulations based on the first three principal components (Fig. 5). We recognize, nonetheless, that the PCA placed four accessions of subpopulation 2 into subpopulation 1 (Fig. 5). This can be explained by differences in the two methods that were applied. The program Structure 2.3.3 along with the method proposed by Evanno et al. (2005) is a Bayesian-based approach (Pritchard et al. 2000), which distributes all the accessions into subpopulations. The PCA method, on the other hand, is essentially based on a few orthogonal principal components, such as the first and second ones, and some accessions might contribute little variation in these components.

The GLM-TP analysis identified two loci significantly associated with stem rust resistance, one on chromosome 1S^Sh^ and the other on 3S^Sh^. The markers found on chromosome 1S^Sh^ genetically map to the same positions as the ones which are closest to the QTL peak for resistance to TPMKC or TRTTF in the 2189 × 1644 RIL population (Fig. 3; Table 1). We identified the gene *Sr-1644-5Sh* in the 2189 × 1644 RIL population, but did not detect any significant marker association by GWAS (with either GLM or GLM-TP). This might be due to lack of closely linked markers in the region around *Sr-1644-5Sh* (Fig. 3) or to a low frequency of *Sr-1644-5Sh* in the diversity panel. The locus on chromosome 3S^Sh^ was not detected in either of the biparental populations. Our results suggest that GWAS can be used as a survey to identify chromosomal regions conferring resistance in a population. However, a major gene with a low allele frequency in the population or minor genes in the population where multiple major genes are present might fall below the detection limit. In contrast, in a biparental population, both major and minor genes (QTL) should be identifiable with adequate population size and genetic linkage map. Therefore, the development of more biparental populations may allow more *Sr* genes to be identified in *Ae. sharonensis*.

Some researchers argue that marker effects for both population structure *Q* and kinship *K* could be over-correcting and might therefore require relaxed *p* value levels such as 0.001 (Pasam et al. 2012; Zegeye et al. 2014) or require ways to correct for background variations (Yu et al. 2006). In this study, the GLM detected seven loci for resistance to stem rust. Excluding the locus on chromosome 1S^Sh^, all other loci were not detected by QTL mapping in the biparental populations. These QTL might reflect true *Sr* genes in other resistant accessions or they might be false discoveries. In contrast, the MLM found no marker significantly associated with stem rust resistance even at the relaxed *p* value of 0.001 (Fig. 6b). Based on the biparental population mapping, we identified two major QTLs/genes (Table 1). The *Sr-1644-1Sh* region is well covered by markers in the diversity panel and some associated markers had very low *p* values in the GLM analysis. *Sr-1644-1Sh* should have been detected, but MLM analysis did not detect this gene (Fig. 6b). This indicates that the structure and kinship effects are probably over-corrected using the MLM in this diversity panel. We also tried to minimize the structure and kinship effects by removing closely related accessions based on cluster analysis. Using GLM-TP, one locus on 1S^Sh^, to where we mapped *Sr-1644-1Sh* in the biparental populations, was detected against both TPMKC and TRTTF, and another locus on 3S^Sh^ was also detected, suggesting that GLM-TP might be the better way for GWAS in terms of reducing false discovery rate and over-correcting.

GWAS is a useful tool which can be used as a survey to identify potential loci of interest in the genome of a species using a germplasm diversity collection from a discrete geographical area to nation- or world-wide. However, the success of GWAS to define traits of interest relies on multiple factors, including population structure, linkage map density, marker quality, minor allele frequency, and the number of genes contributing to the trait. Some genes for a trait may not be identified due to gaps in the linkage map. Markers such as some SSRs, amplify at multiple loci, thus reducing the effectiveness of the detection of association between a marker and a trait. The vast majority of single nucleotide polymorphism (SNP) markers amplify at only one locus. The SNP-based OPA is a high throughput and high quality marker system that can be used to improve genome coverage and linkage density. The consensus OPA genetic linkage map developed in this study should be beneficial for regular gene/QTL mapping in a biparental population. For GWAS, we would advocate the generation of a denser genetic map, such as can be achieved with genotyping-by-sequencing (Li et al. 2015) or the Illumina 9k or 90k SNP platform (Wang et al. 2014).

In this study, we developed the first whole genome genetic linkage map for *Ae. sharonensis* using OPA markers. By QTL mapping, we identified two novel wheat stem rust resistance genes, *Sr-1644-1Sh* and *Sr-1644-5Sh*, in *Ae. sharonensis*. While gene *Sr-1644-1Sh* confers strong resistance to races TTKSK and TPMKC, the gene *Sr-1644-5Sh* confers a high level of resistance to race TRTTF. The genetic isolation of *Sr-1644-1Sh* and *Sr-1644-5Sh* in an otherwise susceptible background and the generation of a genetic linkage map now allows these genes to be targeted for molecular isolation (cloning) through map-based approximation or mutational genomics (MutRenSeq; Steuernagel et al. 2016). Furthermore, the development of linked markers would facilitate their introgression from *Ae. sharonensis* into hexaploid wheat (Millet et al. 2014). *Sr-1644-1Sh* was also detected by GWAS, but *Sr-1644-5Sh* was not detected. This study highlights the importance of QTL mapping in a biparental population to support GWAS as previously demonstrated for downy mildew resistance in Arabidopsis (Nemri et al. 2010).

#### Author contribution statement

GY, NC, BS, PDO, JS, CW, RJ, MJM, IH-P, PG and EM performed experiments. GY and BBHW wrote the manuscript. GY, MJM, HS, JDGJ, ERW, BJS and BBHW contributed to the design of the study.

## Electronic supplementary material

Below is the link to the electronic supplementary material.

Supplementary material 1 (PNG 171 KB)

Supplementary material 2 (PNG 393 KB)

Supplementary material 3 (XLSX 17 KB)

Supplementary material 4 (XLSX 19 KB)

Supplementary material 5 (XLSX 11 KB)

Supplementary material 6 (XLSX 156 KB)

Supplementary material 7 (XLSX 136 KB)

Supplementary material 8 (XLSX 42 KB)

Supplementary material 9 (XLSX 9 KB)

Supplementary material 10 (XLSX 9 KB)
