# Supplementary figures and images for: Discovery and characterization of two new stem rust resistance genes in Aegilops sharonensis
Source: Theor Appl Genet. 2017 Mar 8;130(6):1207–22. doi: 10.1007/s00122-017-2882-8 (PMC5440502; doi:10.1007/s00122-017-2882-8)

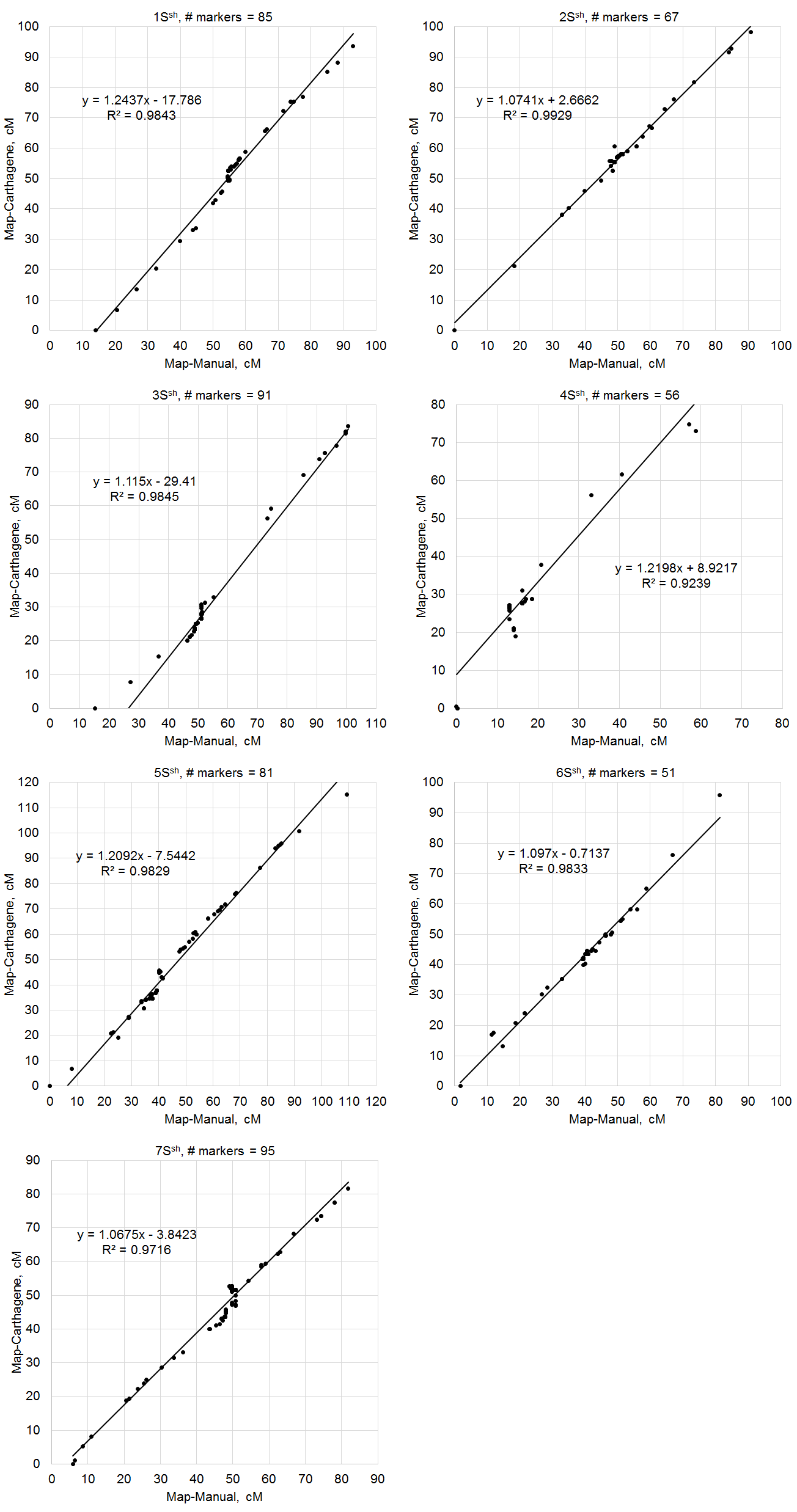

Supplement: Supplementary file 1 — Supplementary material 1 (PNG 171 KB) [file 122_2017_2882_MOESM1_ESM.png]

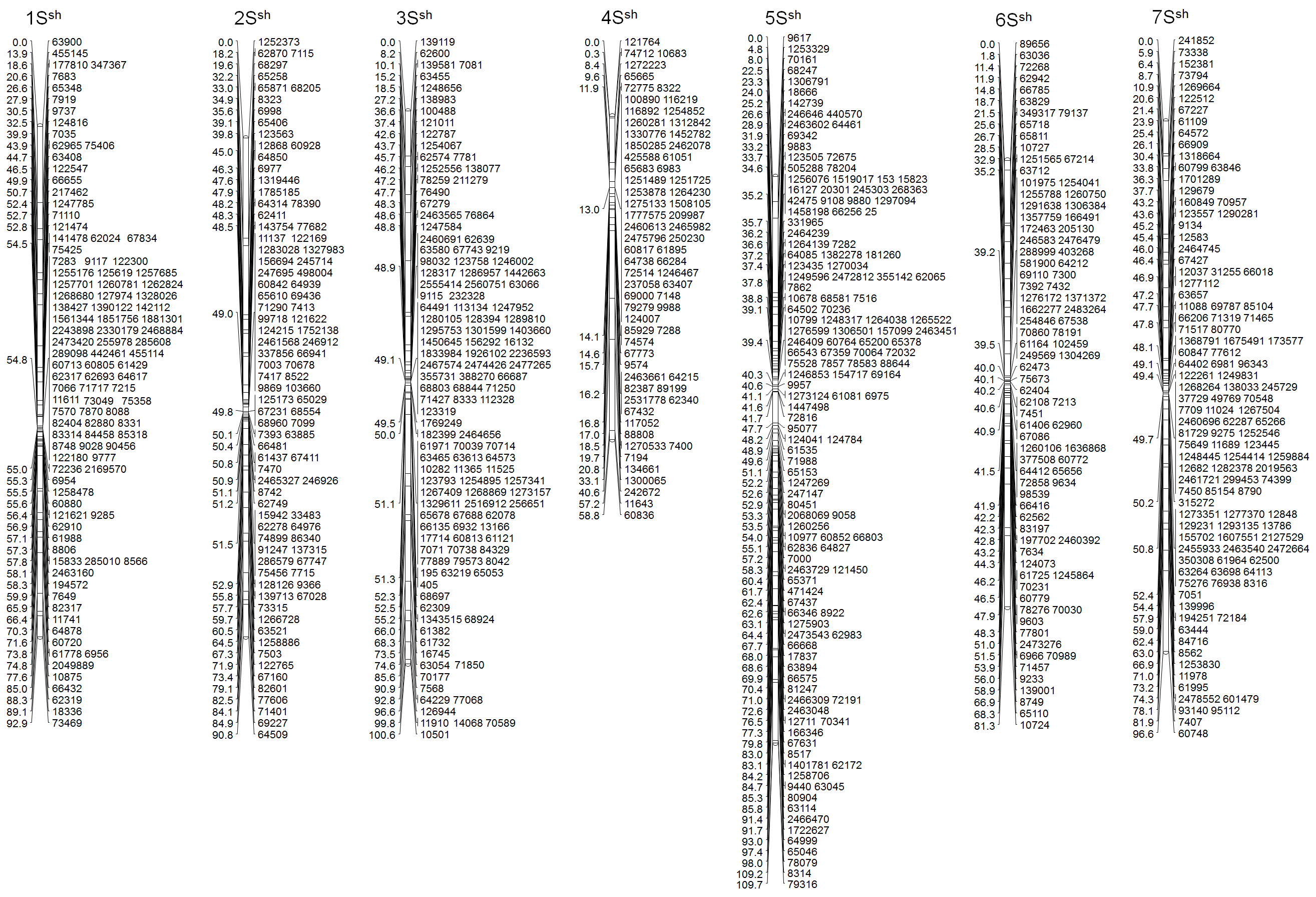

Supplement: Supplementary file 2 — Supplementary material 2 (PNG 393 KB) [file 122_2017_2882_MOESM2_ESM.png]
